# Estimating the Glomerular Filtration Rate in Pediatric Patients With Neurogenic Bladder: A Comparison Between Creatinine- and Cystatin C-Equations

**DOI:** 10.7759/cureus.42337

**Published:** 2023-07-23

**Authors:** Catarina Menezes, Teresa Costa, Catarina Brás, Patrícia Sousa, Ana Mendes, Rosa Amorim, Maria Sameiro Faria, Conceição Mota

**Affiliations:** 1 Pediatrics, Centro Materno Infantil do Norte - Centro Hospitalar Universitário de Santo António, Porto, PRT; 2 Pediatric Nephrology, Centro Materno Infantil do Norte - Centro Hospitalar Universitário de Santo António, Porto, PRT; 3 Nephrology, Hospital Professor Doutor Fernando Fonseca, Amadora, PRT; 4 Pediatrics, Hospitalar da Senhora da Oliveira, Guimarães, PRT; 5 Physical Medicine and Rehabilitation, Centro Hospitalar Universitário de Santo António, Porto, PRT

**Keywords:** neurogenic bladder, egfr cystatin c, egfr creatinine, children's, estimating equations, glomerular filtration rate (gfr)

## Abstract

Background and objective

Patients with neurogenic bladder (NB) are at a higher risk of developing chronic kidney disease (CKD). Due to their lower muscle mass, the estimated glomerular filtration rate (eGFR) based on creatinine (Cr) may be overestimated and delay the diagnosis of renal failure. This study compared eGFR calculated with different equations based on Cr and/or cystatin C (CysC) in children with NB, and the differences between patients with lower muscle mass (underdeveloped lower limbs) and those with independent gait (less muscle depletion).

Methods

We calculated the eGFR in pediatric patients with NB and CKD stages 1 and 2 by using the following equations: Chronic Kidney Disease in Children equation for serum creatinine (CKiD-Cr), CKiD-CysC, CKiD combined-Cr/CysC, Zappitelli-CysC, and Zappitelli combined-Cr/CysC.

Results

We evaluated a total of 47 patients, 74.5% with CKD stage 1, with a median age of 14.1 years. Of these participants, 59.6% had lipo/myelomeningocele. The CKiD-Cr and CysC-based equations led to significantly lower calculated eGFR ​​(p<0.05), specifically CKiD-CysC (p<0.001), Zappitelli-CysC (p<0.001), CKiD-Cr/CysC (p<0.001), and Zappitelli combined-Cr/CysC (p<0.05). When CKiD-CysC was used, 68% of the patients moved to a more advanced CKD stage. In patients without independent gait, with lower muscle mass (55.3%), the median eGFR calculated using the CKiD-Cr and CKiD combined-Cr/CysC equations was significantly higher (p<0.05). However, there were no differences between the two groups when using the other CysC-based equations.

Conclusion

In patients with NB and poor muscle mass, the CKiD-Cr equation may overestimate renal function. CysC-based equations seem more reliable in these patients, especially in those with greater muscular atrophy.

## Introduction

Neurogenic bladder (NB) is defined as a lower urinary tract dysfunction ranging from detrusor underactivity to overactivity, either associated with sphincter detrusor dyssynergia or not, and resulting in inadequate storage capacity, urinary incontinence, and/or ineffective emptying capability. It is caused by a congenital or acquired injury or disease at any level in the central or peripheral nervous system [1-3].

In the pediatric population, the most common cause of NB is spinal dysraphism such as myelomeningocele, lipomeningocele, meningocele, and occult lesions. Other etiologies of NB include sacral agenesis, cerebral palsy, traumatic spinal cord injuries, tumors of the brain or spinal cord, muscular dystrophy, and other neuromuscular diseases [2,4-9]. Most patients with NB have abnormal body morphology with lower muscle mass, especially in the lower limbs, frequently associated with underdeveloped musculature, orthopedic abnormalities, and gait change [2,4-6,8]. The consequences of this neuromuscular disease range from no significant neurological deficit to bladder dysfunction with urinary incontinence, NB-associated kidney disease, and severe neurological motor injury with partial or total paresis. The anatomical level of the spinal lesion and the neurological level are not sufficient to predict the type or severity of lower urinary tract dysfunction because the distal spinal cord (S2-S4) controls the bladder. Patients with low-level (sacral) myelomeningocele may have dysfunctional NB even with normal lower limb function [10,11].

Chronic kidney disease (CKD) is three times more prevalent in patients with NB than in the general population; it affects 25-50% of these patients and is a major cause of morbidity and mortality [4]. Kidney disease is mainly secondary to abnormal bladder function (poor compliance, outlet resistance, and elevated intravesical pressure), which leads to higher pressure in the lower urinary tract with a high risk of developing frequent urinary tract infections, vesicoureteral reflux, and renal scarring and damage. Some patients may progress to kidney failure requiring dialysis or kidney transplantation [1,5,12-14].

It is critical to reach an early diagnosis and to better identify risk factors, in order to prevent kidney disease progression and preserve lifetime kidney function. Thus, accurately measuring kidney function is crucial to identify patients in the early stages of kidney dysfunction and to monitor its progression closely [6,7,14-17]. Evaluation of the glomerular filtration rate (GFR) is essential to assess kidney function. The GFR may be measured directly based on the clearance of exogenous filtration markers such as inulin, or estimated indirectly based on the clearance of endogenous filtration markers such as creatinine (Cr) or cystatin C (CysC). While the first method is considered the gold standard, it has several limitations that make it difficult to perform; thus, it is rarely applied [1,18,19]. In children, the estimated glomerular filtration rate (eGFR) is often calculated by using the Chronic Kidney Disease in Children equation for serum creatinine (CKiD-Cr), also known as the bedside Schwartz equation [19-21].

Cr is a simple and practical renal function marker. It is produced at a fairly constant rate and eliminated mostly by the glomeruli. However, as it is produced from the nonenzymatic degradation of muscle creatine, its level is highly dependent on the patient’s muscle mass, resulting in low sensitivity and accuracy. Besides, it is also affected by age, sex, diet, some drugs, malnutrition, and chronic illness [16,19,20]. On the other hand, CysC is a low-molecular-weight protein produced by all human nucleated cells. It is almost completely filtered by the glomeruli and unaltered by body size and composition, muscle mass, sex, and age [1,5,16,19]. However, CysC determination is not as widely available and is more expensive [20,22].

Several equations based on Cr and/or CysC have been created to improve the accuracy of the eGFR calculation and are widely used: CKiD-CysC, Zappitelli-CysC, CKiD combined-Cr/CysC, and Zappitelli combined-Cr/CysC [19,20]. In patients with NB with reduced muscle mass, determining the eGFR with Cr-based equations may have lower sensitivity and overestimate eGFR, thereby making them potentially unreliable [1,6,15,16,23]. Previous studies have reported significant variability between the eGFR calculated by Cr- and CysC-based equations in adult and pediatric patients with spina bifida or NB. Furthermore, in those studies, the eGFR based on CysC upgraded CKD to a more advanced stage [1,5,15,16,21,23,24]. However, comparative studies of the eGFR between patients with NB and different levels of neurological damage, and consequently with different degrees of muscle mass reduction, are scarce.

In this study, we compared the eGFR calculated by different equations based on Cr and/or CysC in children with NB in the early stages of CKD (stages 1 and 2). We also compared the eGFR calculated with these equations between patients with motor neurological injury (MNI) - patients unable to walk independently, patients with underdeveloped lower limbs and poorer muscle mass - and patients without MNI - with independent gait and with more typically developed lower limb musculature.

This article was previously posted to the Research Square preprint server on 20 April 2023: Menezes C, Costa T, Brás C, et al.: Estimating Glomerular Filtration Rate in Pediatric Patients With Neurogenic Bladder: a comparison between Creatinine- and Cystatin C-Equations. Res Square. DOI: 10.21203/rs.3.rs-2778544/v1.

## Materials and methods

Study design and sample

We conducted a cross-sectional observational retrospective study by analyzing the clinical records of all pediatric patients (aged <18 years) with NB and CKD stages 1 and 2, based on the eGFR calculated with the CKiD-Cr (bedside Schwartz) equation, and followed up between January 2009 and April 2022 at an outpatient multidisciplinary NB clinic of the pediatric nephrology unit of a tertiary pediatric hospital in Portugal.

Variables definition and data collection

We collected data by assessing the electronic clinical files. We collected demographic (age and sex), anthropometric (weight and height), and analytical variables; the latter included serum Cr and CysC and blood urea nitrogen (BUN) values based on the most recent medical consultation and laboratory results carried out during the follow-up. We excluded patients who did not have all the studied variables obtained simultaneously at the most recent medical evaluation in their electronic clinical files. We used height and weight to calculate body mass index (BMI). In wheelchair-bound patients, we used supine length instead of height. We calculated the baseline eGFR value by using CKiD-Cr (bedside Schwartz) equation. All patients fulfilled the Pediatric Kidney Disease: Improving Global Outcomes (KDIGO) CKD criteria. CKD stage 1 is classified as an eGFR level ≥90 mL/min/1.73 m^2^, with evidence of kidney structural damage, and stage 2 is defined as an eGFR level between 60 and 89 mL/min/1.73 m^2^ [25].

We calculated the eGFR (mL/min/1.73 m^2^) for each subject by using the following equations: CKiD-Cr, CKiD-CysC, Zappitelli-CysC, CKiD combined-Cr/CysC, and Zappitelli combined-Cr/CysC (Table 1).

**Table 1:** eGFR calculation formulas based on creatinine and cystatin C SCr: serum creatinine; CKiD-Cr: Chronic Kidney Disease in Children equation for serum creatinine; CysC: cystatin C; BUN: blood urea nitrogen

Formula	Equation
CKiD-Cr 2009 (Schwartz bedside)	eGFR = 0.413 x [Height (cm)/SCr (mg/dL)]
CKiD-CysC 2012	eGFR = 70.69 x [CysC^(-0.931)]
CKiD-Cr/CysC (Schwartz combined)	eGFR = 39.8 x [(Height (m)/SCr)^0.456] x [(1,8/CysC)^0.418] x [(30/BUN)^0.079] x [(Height (m)/1.4)^0.179]
Zappitelli-CysC	eGFR = 75.94/CysC
Zappitelli combined-Cr/CysC	eGFR = [507.76^(0.003 x Height (cm)] / [(CysC^0.635) x (SCr x 88.4)^0.547)]

We classified patients with NB into two groups: one group of patients with MNI - those without independent gait and with more significant muscle atrophy (including paraparesis, paraplegia, use of orthosis or wheelchair); and a second group including children without MNI - with independent gait and less muscle mass reduction.

Ethical consideration

This research complied with all the relevant national regulations and institutional policies and is in accordance with the tenets of the Declaration of Helsinki. This study was approved by the Ethics Committee of Centro Hospitalar Universitário de Santo António and Institute of Biomedical Sciences Abel Salazar - 2022-289 (227-DEFI/245-CE).

Statistical analysis

We used SPSS Statistics 28.0 (IBM Corp., Armonk, NY) for statistical analysis. Continuous variables had an asymmetrical distribution and are expressed as median and the interquartile range. We compared independent continuous variables with the Mann-Whitney U test and paired variables with the Wilcoxon test. We evaluated differences in the distribution of categorical variables with the chi-square test. All p-values were two-sided and we consideredp<0.05 to be statistically significant.

## Results

We included 47 patients in our study. Of these, 24 (51.1%) were female and 23 (48.9%) were male. In 28 patients (59.6%), NB was due to lipo/myelomeningocele. The causes of NB in the other patients were as follows: muscular dystrophy in five, cerebral palsy in three, sacral agenesis in three, tumors of the spinal cord in two, syringomyelia in one, traumatic spinal cord injury in one, and other neuromuscular diseases in five. As per their latest medical consultation, the patients had a median age of 14.1 (8.9-16.9) years, a median weight of 38 (22.0-55.8) kg, a median height of 142 (120-148) cm, and a median BMI of 20.5 (15.5-26.5) kg/m^2^ (Table 2). Regarding the CKD stage, 35 (74.5%) were classified as stage 1 and 12 (25.5%) as stage 2.

**Table 2:** Demographic, anthropometric, and analytical characteristics and CKD staging of the sample BMI: body mass index; BUN: blood urea nitrogen; CKD: chronic kidney disease

Variables	Study sample	Group with motor neurological injury	Group without motor neurological injury
Total, n (%)	47 (100%)	26 (55.3%)	21 (44.7%)
Age, years, median (25^th^-75^th^ percentile)	14.1 (8.9–16.9)	13.3 (8.9–16.6)	14.1 (9.7–17.3)
Sex, female, n (%)	24 (51.1%)	11 (42.3%)	13 (61.9%)
Weight, Kg, median (25^th^-75^th^ percentile)	38 (22–55.8)	35.8 (21–53.7)	43 (26.5–66)
Height, cm, median (25^th^-75^th^ percentile)	142 (120–148)	137.5 (113.3–147)	145.5 (126.5–158)
BMI, Kg/m^2^, median (25^th^-75^th^ percentile)	20.5 (15.5–26.5)	21.2 (15.3–26.2)	20.5 (15.4–29.3)
CKD stage 1, n (%)	35 (74.5%)	23 (88.5%)	12 (57.1%)
CKD stage 2, n (%)	12 (25.5%)	3 (11.5%)	9 (42.9%)
Creatinine (Cr), mg/dL, median (25^th^-75^th^ percentile)	0.52 (0.42–0.67)	0.46 (0.36–0.53)	0.67 (0.5–0.9)
Cystatin C (CysC), mg/L, median (25^th^-75^th^ percentile)	0.91 (0.77–1.2)	0.89 (0.8–1.2)	0.95 (0.75–1.2)
BUN, mg/dL, median (25^th^-75^th^ percentile)	15.4 (12.6–20.1)	14 (12.4–18.3)	15.9 (13.5–21.5)

The cohort had a median serum Cr value of 0.52 (0.42-0.67) mg/dL, median serum CysC value of 0.91 (0.77-1.2) mg/L, and median BUN value of 15.4 (12.6-20.1) mg/dL (Table 2). For the overall population, the median eGFR (mL/min/1.73 m2) calculated with each equation was as follows - CKiD-Cr: 110.36 (86.24-129.31); CKiD-CysC: 76.94 (59.65-89.73); Zappitelli-CysC: 83.18 (63.28-98.11); CKiD combined-Cr/CysC: 86.60 (65.76-101.33); and Zappitelli combined-Cr/CysC: 103.42 (72.71-120.50) (Table 3). When compared with the CKiD-Cr (bedside Schwartz) equation, each CysC-based equation showed significantly lower eGFR values ​​(p<0.05), as follows: CKiD-CysC (p<0.001), Zappitelli-CysC (p<0.001), CKiD combined-Cr/CysC (p<0.001) and Zappitelli combined-Cr/CysC (p<0.05) (Table 3). When we used the CKiD-CysC equation to calculate eGFR, the CKD classification changed for 32 (68%) patients to more advanced stages: 21 (44.7%) patients would be classified as stage 2 and 11 (23.4%) as stage 3. In the group of patients with MNI and greater muscle depletion, 21 (80.7%) were upstaged.

**Table 3:** Comparison between the median CKiD-Cr (Schwartz bedside) and the other eGFR formulas based on Cr and/or CysC CKiD-Cr: Chronic Kidney Disease in Children equation for serum creatinine; CysC: cystatin C; eGFR: estimated glomerular filtration rate

eGFR, mL/min/1.73m^2^	Study sample	P-value
CKiD-Cr (Schwartz bedside), median (25^th^-75^th^ percentile)	110.36 (86.24–129.31)	
CKiD-Cr/Cys (Schwartz combined), median (25^th^-75^th^ percentile)	86.60 (65.76–101.33)	<0.001
CKiD-CysC, median (25^th^-75^th^ percentile)	76.94 (59.65–89.73)	<0.001
Zappitelli-CysC, median (25^th^-75^th^ percentile)	83.18 (63.28–98.11)	<0.001
Zappitelli combined-Cr/CysC, median (25^th^-75^th^ percentile)	103.42 (72.71–120.50)	0.002

When we classified patients with NB into two distinct groups, 26 (55.3%) had MNI while 21 (44.7%) did not. We compared the median Cr and CysC values between the patients with MNI (who used a wheelchair or orthotics and had lower muscle mass and underdeveloped lower limbs) and the patients without MNI (who were ambulatory and had more typically developed lower limb musculature). The patients with MNI had a significantly lower median Cr value [0.46 (0.36-0.53) mg/dL] compared to the patients without MNI [0.67 (0.5-0.9)] mg/dL, (p<0.001). However, the median CysC value was similar in the patients with MNI [0.89 (0.8-1.2) mg/L] and those without MNI [0.95 (0.75-1.2) mg/L; p = 0.764) (Table 4).

**Table 4:** Comparison of median creatinine, cystatin C, and creatinine- and cystatin C-eGFR using different formulas between the group with motor neurological injury and that without motor neurological injury eGFR: estimated glomerular filtration rate

	Group with motor neurological injury (n = 26)	Group without motor neurological injury (n = 21)	P-value
Creatinine, mg/dL, median (25^th^-75^th^ percentile)	0.46 (0.36–0.53)	0.67 (0.5–0.9)	<0.001
Cystatin C, mg/L, median (25^th^-75^th^ percentile)	0.89 (0.8–1.2)	0.95 (0.75–1.2)	0.764
eGFR, mL/min/1.73m^2^			
CKiD-Cr (Schwartz bedside), median (25^th^-75^th^ percentile)	118.52 (101.21–152.14)	91.23 (64.25–112.16)	<0.001
CKiD-Cr/Cys (Schwartz combined), median (25^th^-75^th^ percentile)	91.97 (72.82–115.01)	82.80 (63.59–88.60)	0.032
CKiD-CysC, median (25^th^-75^th^ percentile)	78.38 (59.65–87.01)	74.07 (59.42–92.46)	0.764
Zappitelli-CysC, median (25^th^-75^th^ percentile)	84.85 (63.28–94.93)	79.85 (63.02–101.36)	0.764
Zappitelli combined-Cr/CysC, median (25^th^-75^th^ percentile)	107.21 (79.66–142.16)	88.39 (65.26–110.43)	0.063

The median eGFR calculated using the CKiD-Cr and CKiD-Cr/CysC equations was significantly higher (p<0.05) in the patients with MNI compared with the patients without MNI [118.52 (101.21-152.14) and 91.97 (72.82-115.01) vs. 91.23 (64.25-112.16) and 82.80 (63.59-88.60) mL/min/1.73 m^2^] (Table 4). However, there were no significant differences (p>0.05) between the two groups when comparing the median eGFR using the CKiD-CysC equation [78.38 (59.65-87.01) vs. 74.08 (59.42-92.46) mL/min/1.73 m^2^] (Table 4).

## Discussion

The importance of early diagnosis of kidney disease in patients with NB and the risk of CKD progression is paramount. An early diagnosis enables the implementation of the best therapeutic plan and the optimal follow-up strategy to prevent progressive kidney damage. Earlier detection of CKD in this population also allows a timely referral to pediatric nephrology care and closer monitoring [16,26]. Regular direct GFR measurement in children with NB is impractical and 24-hour urine collection may be inaccurate in these patients, particularly in those with incontinence.

The eGFR calculated with the CKiD-Cr (bedside Schwartz) equation is the most commonly used method to assess kidney function in children. However, the reduced muscle mass in patients with NB turns Cr into a less accurate marker, as reported in several previous studies [5,15,16,22,23,27]. On the other hand, although combined Cr/CysC-based equations have demonstrated better accuracy in estimating GFR in children with CKD [19,28], they may show worse performance than CysC-based equations in pediatric patients with NB [15,16,29]. Additionally, Cr-based equations employed to estimate the GFR require an accurate height measurement, which can be difficult to obtain in non-ambulatory patients with NB and paretic, underdeveloped, and often spastic lower limbs [13].

The National Institute for Health and Care Excellence guidelines (UK) recommend not using serum Cr and Cr-based eGFR equations alone to monitor renal function in patients with NB [30]. Our study corroborates this by demonstrating a significantly higher median eGFR value with the CKiD-Cr equation when compared with the CysC-based equations. Our results are also in agreement with urological guidelines for the care and management of people with spina bifida from the Spina Bifida Association, which recommends the use of serum CysC or a nuclear medicine GFR test [13].

Dangle et al. [16] published a prospective single-center study involving 131 pediatric patients with myelomeningocele and NB. They used the CKiD-Cr and Zappitelli-CysC equations to evaluate kidney function in the early stage of the disease (they excluded patients with CKD stage >2). They reported a lower eGFR with the CysC equation when compared to the CKiD-Cr equation. Using the Zappitelli-CysC equation, CKD was upgraded to stage >2 in 26% of these children. They concluded that the CysC-based equation is more sensitive in detecting early CKD in this high-risk population [16]. In our study, in the overall sample, the median eGFR calculated with all the CysC-based equations assessed, namely CKiD-CysC, Zappitelli-CysC, CKiD combined-Cr/CysC, and Zappitelli combined-Cr/CysC, showed significantly lower eGFR values compared with the CKiD-Cr equation, which is in line with the results of Dangle et al. [16]. On the other hand, when we used the CKiD-CysC equation to calculate the eGFR, 68% of our patients would have been classified as having more advanced CKD stages: 48.9% with stage 2 and 27.7% with stage 3. This finding is also consistent with the study by Dangle et al. [16], and indeed even more pronounced, especially in the group of patients with greater muscle atrophy.

Zhou et al. [15] conducted an interesting prospective study involving 458 children (2-16 years old) with NB to evaluate the performance of different equations to estimate GFR. They regarded the GFR measured with the technetium 99m-diethylene triamine penta-acetic acid (99mTc-DTPA) dual plasma sample clearance method as the reference standard. They compared the CKiD-Cr, CKiD-CysC, and CKiD combined-Cr/CysC equations to the reference standard GFR. The correlation between the Cr-based equation and 99mTc-DTPA clearance method was low (r = 0.648); the CysC-based equations had much stronger correlations (CKiD-CysC, r = 0.891; CKiD combined-Cr/CysC, r = 0.879). Their findings showed that the CKiD-Cr equation overestimated GFR and that CysC-based equations could be more sensitive in evaluating kidney function in children with NB in the initial stages of kidney disease, and hence they should be recommended in children with NB to estimate GFR [15]. Although we did not confirm the accuracy of Cr- and/or CysC-based eGFR equations by using a reference method in our study, our results also showed that combined Cr/CysC and CysC equations produced significantly lower eGFR values compared with the CKiD-Cr equation. The fact that our findings are consistent with the study by Zhou et al. [15], who validated their results by comparison with a reference standard, reinforces our view that CysC-based equations may be more reliable in these patients.

Our results are also in line with those of Morgan et al. [29], who evaluated children with spina bifida. They reported a significant and higher correlation between 99mTc-DTPA GFR and eGFR calculated by a CysC-based equation than that of eGFR calculated by the Cr-based beside Schwartz equation (that correlation was not significant).

Morrow et al. [5] compared Cr- and CysC-based eGFR calculations in adult patients with spina bifida and different MNI levels and reported a significantly higher mean eGFR based on Cr in patients with thoracic myelomeningocele compared with patients with sacral level. There was no difference in the mean eGFR based on CysC between patients with thoracic and sacral myelomeningocele. In addition, the difference between the mean eGFR based on Cr and the mean eGFR based on CysC was significantly higher in patients with a thoracic-level injury. Their study recommends using CysC to monitor kidney function in patients with spina bifida, particularly in those with thoracic-level lesions, who tend to have lower muscle mass [5]. We also noted significantly higher median eGFR values in patients with MNI, who have a lower muscle mass, when using the CKiD-Cr and CKiD combined-Cr/CysC equations when compared with the other group of patients without MNI and with a more typical muscle mass. Nonetheless, we found no significant differences between the two groups when comparing the median eGFR calculated with the CysC-based equation, namely the CKiD-CysC equation. These findings indicate that CysC may be a more sensitive and reliable marker to assess kidney function in the early stages of kidney disease in this population, especially in patients with more significant muscle depletion.

Our study has a few limitations. As it was a retrospective study, we did not confirm the accuracy of Cr- and/or CysC-based eGFR calculation by using a clearance method that measures exogenous filtration markers, which is the gold standard, or by using nuclear-medicine-based GFR evaluation or 24-hour urine Cr and/or urea clearance. Additionally, this was a single-center study, and hence the generalizability of the results might be limited. Despite these limitations, to our knowledge, this is the first Portugal-based observational retrospective study comparing GFR estimated based on Cr and CysC in pediatric patients with NB-associated kidney disease as well as the differences between patients with and without MNI, and with very different degrees of muscle mass reduction.

## Conclusions

As creatinine is highly dependent on patient muscle mass, Cr-based formulas may overestimate eGFR in children with NB, especially in those with greater muscle atrophy. This may delay CKD diagnosis or even prevent a correct disease staging, leading to several therapeutic and prognostic implications. Hence, since CysC is unaltered by body size and composition, and muscle mass, CysC-based formulas may be a more reliable method to assess kidney function in these patients. Further studies need to be conducted in the pediatric population with NB, including multicentric studies with a larger number of patients, which compare Cr- and CysC-eGFR calculations with a GFR standard measuring method such as nuclear medicine-based GFR evaluation or 24-hour urine Cr and/or urea clearances. It would also be important to compare eGFR calculated with different formulas based on Cr and/or CysC in children with NB and in a control group of children without NB and muscle depletion, to identify a more accurate tool to estimate kidney function in these patients and to support consensus recommendations.
